# Determining the critical buckling load of locally stiffened U-shaped steel sheet pile using dynamic correlation coefficient method

**DOI:** 10.1038/s41598-022-17070-w

**Published:** 2022-07-28

**Authors:** Caihua Shen, Hansen Yu, Xiaojun Wang, Kai Tang, Cornelia Asiedu-Kwakyewaa, Hanyi Zhang

**Affiliations:** 1 College of Civil and Transportation Engineering, Hohai University, Nanjing, 210098 People’s Republic of China; 2 Hangzhou Communications Investment Construction Management and Project Management Ltd, Zhejiang, 310024 China; 3 Poly Changda Overseas Engineering Co., Ltd, Guangzhou, 51000 China

**Keywords:** Civil engineering, Structural materials, Theory and computation

## Abstract

U-Shaped Steel Sheet Piles (USSSP) have buckling and bending problems during construction. In this research, the structural measure of stiffened plates applied along the longitudinal direction of USSSP is analyzed and proposed. A theoretical calculation method of stiffening effect is proposed based on the dynamic correlation coefficient method and the formula for the critical buckling load of locally stiffened USSSP is derived. A parametric analysis of the number, width, and arrangement scheme of the stiffened plates on the USSSP with different lengths is examined. The empirical formula derived for the total stiffening area and the critical load of the axial compression members of the USSSP is compared with practical results from an engineering case study. The results show a quadratic function under the condition of arranging the different number of stiffened plates at equal intervals, which is accurate in deriving the critical load values USSSP after local stiffening, and provide a theoretical basis for the optimization design of local reinforcement of USSSP axial compression members in engineering projects.

## Introduction

U-shaped steel pile is an axial compressive member with open uniaxial symmetry, which is prone to bending and buckling phenomena during insertion. The insertion and driving of USSSP in engineering has always been a challenge in construction. In recent years, many researchers have mainly focused on the flexural performance of USSSP. Some scholars have studied the relationship between the pile end lock and the flexural stiffness of steel sheet piles. A study proposed that the flexural stiffness of the USSSP wall section increases nonlinearly with the increase of the ratio of the resultant frictional force and the bending moment from the section to the lock^1^. The friction coefficient of the lock is very critical for the stiffness in steel sheet piles^2^. In addition, the flexural stiffness of USSSP is positively correlated with the stiffness of the lock^3^. The improvement of the flexural stiffness of steel sheet piles has a protective effect on the lock ^4^. While considering the lock friction through comparative experiments found that adding sand in the interlocking of USSSP can significantly improve the flexural rigidity of steel sheet piles ^5^. A reduction of flexural strength of steel sheet piles can be achieved by combining soil-structure interactions during the piling process^6^. Also, a finite element model of steel sheet piles was developed considering the interaction between the locks, and concluded that the stiffness reduction factor in the actual engineering design should not be less than 0.5, otherwise the impact of the locks on the support structure of the steel sheet pile will be exaggerated^7^. A study proposed through a test comparison that the cross-sectional modulus value of the steel plate pile when the lock is free is less than the cross-sectional modulus value at the time of the lock welding, therefore welding is beneficial to improving the internal force of the steel plate piles^8^. Meanwhile, it was recommended that the USSSP under the action of the composite force should be welded to the lock, saving steel and lowering the cost significantly^9^. Weld seams can effectively limit the relative slippage of USSSP composite specimens and effectively cooperate with adjacent steel sheet pile specimens to work together^10^. Some scholars have simulated the construction of steel sheet piles to build corresponding models. Drawing a conclusion, the higher axial resistance in the corner area forces the deformation of the section by simulating the ejection of U-shaped and Z-shaped sheet piles^11^. In determining the RMA value through the finite element model, an optimization strategy for the design of the U-shaped steel sheet pile support structure was proposed^12^. A numerical model for predicting the bending stress and critical buckling force of USSSP from bending was proposed^13^. An empirical formula was established to approximately predict the stratum strength by quantitatively analyzing the pile driving curves pattern of the three kinds of construction methods of USSSP^14^. There are also scholars who studied the detection and corrosion of steel sheet piles, amongst which includes a detection method for quantifying the life cycle cost and environmental impact of steel sheet pile^15^. Also early perforation in the web and corner regions of U-shaped steel sheet piles subjected to accelerated corrosion is associated with local segregation and differences in metal composition^16^. In addition, the pass system for rolling USSSP using Pro/E software was designed to simulate the rolls and rolled parts^17^. Also, the rationality of the pass system was verified by simulating the entire rolling process with the DERORM software. Moreover, there was a redesign of the pass system of the USSSP using Pro/E software to model, imported it into DEFORM-3, a finite element analysis software, then simulated and analyzed the newly build pass system to ensure its logic^18^. KIMURA^19–24^ proposed the possibility of bending and buckling of slender piles in liquefied soil, clarified the buckling behaviour of steel piles with rotating springs in liquefied soil during earthquakes, estimated the ultimate strength of slender piles in liquefied soil under vertical and horizontal loads, and described the collapse mechanism and the ultimate strength of piles produced by dynamic buckling of piles in liquefied soil through centrifuge tests. In general, there are many achievements in the study of the buckling and flexural performance of USSSP using finite element and experimental methods. However, there are few theoretical studies and calculation methods for strengthening the bending and buckling of USSSP.

## Derivation of the calculation formula for the critical buckling load of locally stiffened USSSP based on the dynamic correlation coefficient method

**Figure 1:**
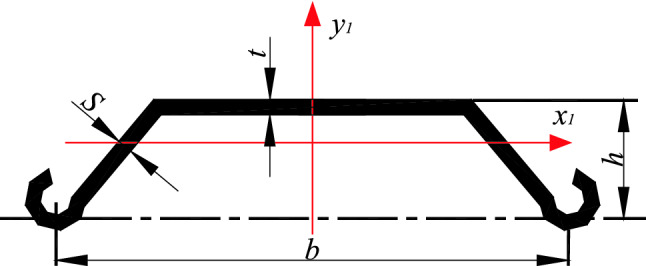
Unstiffened U-shaped steel sheet pile.

**Figure 2:**
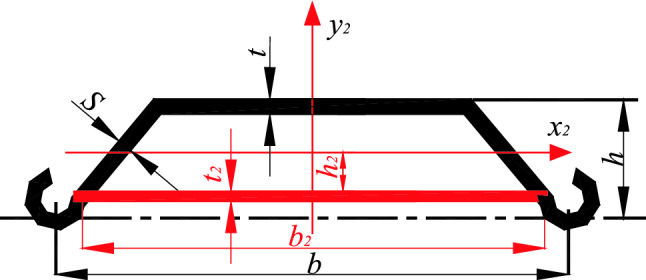
U-shaped steel sheet pile after stiffening.

At present, there is no theoretical calculation method for the ultimate load of USSSP with stiffened plates in the current steel structural design codes. In this paper, the influence of the size and the stiffening position of the stiffened plate is considered during the stiffening arrangement. The dynamic correlation coefficient method is proposed, and the calculation method of the critical buckling load of the stiffened member is derived. The specific calculation method is as follows:

Firstly considering the standardized stability calculation method, take the center coordinates of the standard U-shaped steel sheet pile (referred to as the standard member) as the coordinate origin, and establish the *x*_1_–*y*_1_ rectangular coordinate system (as shown in Fig. 1). Secondly, take the center coordinates of the fully stiffened plate (referred to as the full-length stiffener) arranged longitudinally along the length of the axial compression member of the U-shaped steel sheet pile as the coordinate origin and establish the *x*_2_–*y*_2_ rectangular coordinate system (as shown in Fig. 2). The corresponding critical load values under these two limit states is calculated. The effect of local stiffening on the axial compression member of the fully stiffened U-shaped steel sheet pile is then simplified as a linear function. Finally, the stiffening effect of the stiffened plate is converted into a position function, the dynamic correlation coefficient is obtained, and the critical load value of the U-shaped steel sheet pile after local stiffening is also achieved.

According to the actual construction requirements, the AU series of standard steel sheet piles are selected, and their cross-sectional parameters can be referred to the Steel Sheet Pile Engineering Manual ^25^.

### Stability calculation of standard USSSP and full-length stiffened USSSP

(1) According to the Code for Design of Steel Structures (specification GB50017-2013) ^26^, the critical buckling load of the standard U-shaped steel sheet pile $N_{1\max }$ is calculated as follows:

1 $$N1\max = \phi \cdot A \cdot f$$

where *A* is the total cross-sectional area of the member equal to the cross-sectional area of the USSSP in cm^2^; *f* is the design value of the compressive strength of the steel, N/mm^2^ and $\phi$ is the stability coefficient.

The solution method of the stability coefficient $\phi$ is as follows:

2 $$\begin{aligned} & {\text{When}}\;\overline{\lambda 0} = \frac{\lambda }{\pi }\sqrt{\frac{fy}{E}} \le 0.215; \\ & \phi = 1 - 0.65\overline{\lambda 0}^{2} \\ & {\text{When}}\;\overline{\lambda 0} > 0.215; \\ & \phi = \frac{1}{{2\overline{\lambda 0}^{2} }}\left[ {\left( {0.965 + 0.3\overline{\lambda 0} + \overline{\lambda 0}^{2} } \right) - \sqrt {\left( {0.965 + 0.3\overline{\lambda 0} + \overline{\lambda 0}^{2} } \right)^{2} - 4\overline{\lambda 0}^{2} } } \right] \\ \end{aligned}$$

where $\overline{\lambda 0}$ is the calculated relative slenderness ratio, $\overline{\lambda 0} = \frac{\lambda }{\pi }\sqrt{\frac{fy}{E}}$; $\lambda$ is the slenderness ratio which is given as $\lambda = \frac{\mu l}{i}$, where *i* is the radius of gyration cm; $i = \sqrt{\frac{I}{A}}$, *I* is the moment of inertia of the member, cm^4^; $\mu$ is the calculated length coefficient, *l* is the length of the component, m; $fy$ is the yield strength of the steel, N/mm^2^; *E* is the elastic modulus of the steel, GPa.

(2) The critical load of the axial compression members of the USSSP with longitudinal fully configured stiffeners $N2\max$ is calculated as follows:

3 $$N2\max = \phi \cdot A \cdot f$$

$A = S1 + S2$, where *S*_1_ is the cross-sectional area of the standard USSSP, cm^2^; *S*_2_ is the cross-sectional area of the stiffened plate, cm^2^; *f* is the design value of the compressive strength of the steel, kN/mm^2^; the solution method of the stability coefficient $\phi$ as shown in (Eq. 2), where $I_{y} = I_{y1} + S_{1} \cdot h_{1}^{2} + I_{y2} + S_{2} \cdot h_{2}^{2}$, *I*_*y*1_ is the moment of inertia of the standard USSSP, cm^4^; *I*_*y*2_ is the moment of inertia of the stiffened plate, cm^4^; *h*_1_ is the distance between the centroid of the standard USSSP and the centroid of stiffened member, cm; *h*_2_ is the distance between the centroid of the stiffened plate and the centroid of the stiffened member, cm. When the stiffened plate is made of 9.1 mm steel plate, and the distance from the axis of the steel plate to the centroid of the U-shaped steel sheet pile is 10 cm, the moment of inertia of the combined body is calculated as shown in Table 1.

**Table 1:** Geometric parameters and moment of inertia of USSSP assembly after longitudinal stiffening.

Project	Base width /cm	Distance from the centroid to bottom /cm	Distance from steel sheet pile centroid /cm	Thickness /cm	Area /cm^2^	Moment of inertia /cm^4^	Radius of gyration /cm
U-shaped steel sheet pile	75	13.53	0	1.05	112.7	8760	8.82
Stiffener board	70.92	3.53	10	0.91	64.54	4.45	0.26
Full penetration stiffened U-shaped steel sheet piles		9.89	3.64		177.24	12,868.20	8.52

Q235 steel grade is selected as the stiffened steel plate with *f* = 215 MPa, *E* = 206 GPa in Table 1 The critical load calculation of the standard components with lengths of 10 m, 20 m and 30 m respectively and the full-length stiffening conditions are shown in Table 2.

**Table 2:** Critical load of standard and Stiffened USSSP.

Project	*l/*m	*N*_*cr*_/kN	*λ*	$\overline{{\lambda_{0} }}$	*φ*	*N*_*max*_/kN
U-shaped steel sheet pile	10	1781.03	113.43	1.166	0.502	1216.00
	20	445.26	226.85	2.333	0.160	388.70
	30	197.89	340.28	3.499	0.075	181.62
Full-length stiffened U-shaped steel sheet piles	10	2616.9	117.37	1.21	0.480	1828.16
	20	654.2	234.74	2.41	0.151	574.26
	30	290.8	352.11	3.62	0.070	267.68

### The principle of dynamic correlation coefficient method for USSSP stiffening

**Figure 3:**
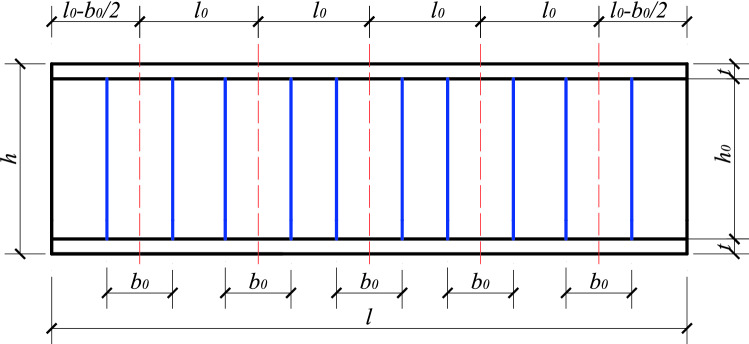
Diagram of longitudinal stiffening of USSSP.

The method of longitudinal installation of USSSP is rarely used because of the large quantity of steel, cost and high frictional resistance during construction. In practical projects, the local stiffening of the stiffening ribs plates (width of $b_{0}$ as shown in Fig. 3) are mostly used to improve the construction stability of USSSP. The dynamic correlation coefficient method is utilized for the analysis considering the position and size effect of the stiffened plate. This method is to comprehensively reflect the stiffening effect of the plate through a dynamic correlation coefficient *δ* relative to the longitudinal penetration under the different plate width *b*_0_ and plate spacing *l*_0_ which has a different stiffness contribution to the entire axial compression member, thereby increasing the critical buckling load of member to varying degrees. The calculation formula of the critical buckling load of the locally stiffened member is as follows:

4 $$N_{\max } = \delta (N_{2\max } - N_{1\max } ) + N_{1\max } = (1 - \delta ) \cdot N_{1\max } + \delta \cdot N_{2\max }$$

#### Basic assumptions

According to the actual situation, the stiffening plates are usually arranged symmetrically, and the basic assumptions of the dynamic correlation coefficient method for solving the critical buckling load of USSSP under partial stiffening conditions are as follows:

1. Assuming that the shape and size of each stiffened plates are the same, the stiffeners are symmetrically arranged, the dynamic correlation coefficient *δ* of each stiffened plate is related to the number of the stiffened plates *n*, the width *b*_0_ of the stiffened plates and the spacing *l*_0_ of the stiffened plates. Every single plate corresponds to a position function, and the dynamic correlation coefficient *δ* that can comprehensively reflect the stiffening effect is obtained through the calculation of the position function.
2. Assuming that multiple stiffening plates are arranged, the total stiffening effect is the combination of the stiffening effects of each plate, and the corresponding overall position function is the envelope function, which is composed of the position functions corresponding to all single plates.
3. Assuming that local stiffening is adopted and the spacing between the stiffened plates is *b*_0_*,* the critical load calculated ($n \cdot b0 = l$) is equal to the critical buckling load calculated by the specification for full-length stiffening.

####  Definition and calculation method of position function

(1) Layout of the Position function of a stiffened plate.

**Figure 4:**
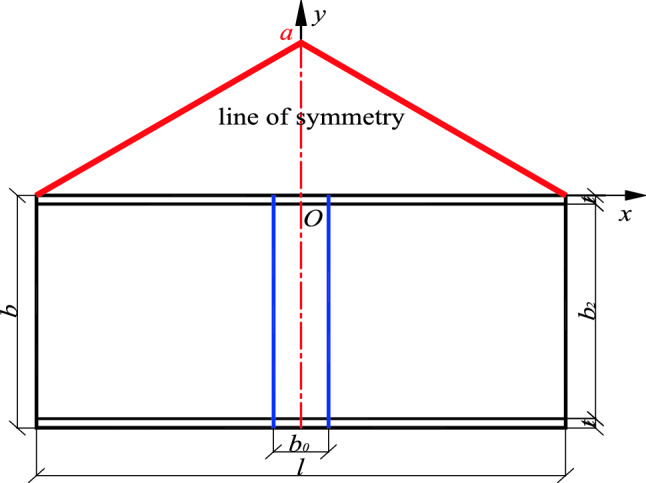
Position function of a stiffened plate.

When arranging a stiffened plate, the stiffening effect ranges between the left and right boundaries of the component. The position function is shown in Fig. 4. The red solid line is the position function corresponding to the stiffened plate, the domain is $\left[ { - \frac{l}{2},\frac{l}{2}} \right]$. There is a maximum value *a* at the center line of the stiffened range, that is the symmetry of the component, and the specific function expression is:

5 $$y = f(x) = \left\{ {\begin{array}{*{20}l} {\frac{2a}{l}x + a} \hfill &\quad { - \frac{l}{2} \le x \le 0} \hfill \\ { - \frac{2a}{l}x + a} \hfill &\quad {0 < x \le \frac{l}{2}} \hfill \\ \end{array} } \right.$$

The position function is a segmented function representing the stiffening effect of the stiffened plate on the standard member, reflecting the size effect and position effect of the stiffened plate. Also, it defines the domain as the scope of the stiffened plate and has a maximum value *a* at its symmetry.

(2) Position function of n (n ≥ 2) stiffened plates.

**Figure 5:**
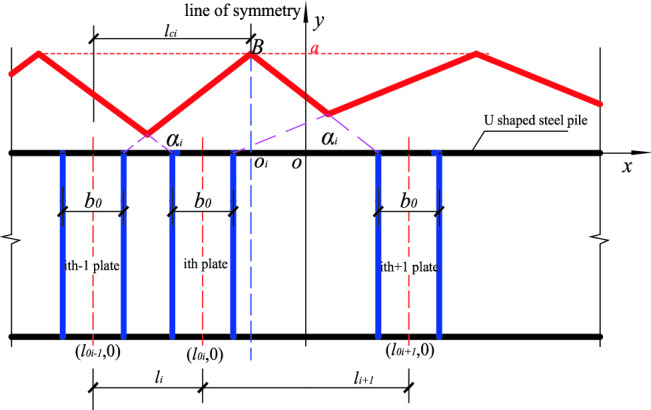
Position function of the inner envelope of n stiffened plates.

**Figure 6:**
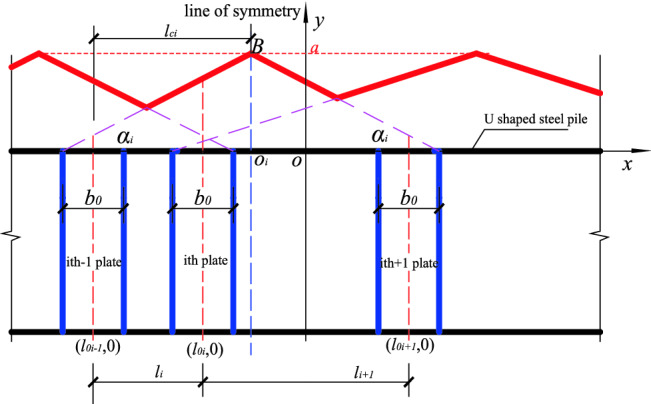
Position function of the outer envelope of n stiffened plates.

When n (n ≥ 2) stiffened plates are arbitrarily arranged, each stiffened plate corresponds to a position function, and the position function of any stiffened plate on the same component has the same maximum value *a* at the center line of the acting range of the stiffened plate. The position functions of adjacent stiffened plates has overlapping parts, the larger *yi* is taken as the overall position function value, and the resulting envelope function is the overall position function of *n* stiffened plates (Fig. 5, shown by the red solid line in Fig. 6). The overall position function is divided into two kinds of envelopes, inner envelope and outer envelope. When stiffening effect of the stiffened plate ranges between the inner edges of the adjacent stiffened plates, the overall position function is the inner envelope. Also, when the stiffening effect of the stiffened plate ranges between the outer edges of the adjacent stiffened plate, the overall position function is said to be the outer envelope as shown in Figs. 5 and 6. *BO*_*i*_ is the line of symmetry/center line of the position function, and it is related to the *i* − 1 (or *i* + 1) while the distance between the line of symmetry and the axis of the stiffened plates is denoted as *l*_*ci*_, and the distances between the axis of the two adjacent stiffened plates are *l*_*i*_ and *l*_*i*+1_ respectively. The following calculation formulas are all taken as an example of the position function of the inner envelope. The definition domain of the corresponding position function to the *i*th stiffened plate is defined between the edges of two adjacent plates as shown in Fig. 5.

The domain definition is $\left[ {l0i - 1 + \frac{b0}{2},l0i + 1 - \frac{b0}{2}} \right]$, at the center line (that is $x = \frac{l0i - 1 + loi + 1}{2}$, shown by the blue dotted line), has a maximum value *a*, and the position function of the *i*th stiffened plate is expressed as:

6 $$y = f(x) = \left\{ {\begin{array}{*{20}c} {\frac{{2a}}{{l_{{0i + 1}} - l_{{0i - 1}} - b_{0} }}x - \frac{{2a\left( {l_{{0i - 1}} + \frac{{b_{0} }}{2}} \right)}}{{l_{{0i + 1}} - l_{{0i - 1}} - b_{0} }},l_{{0i - 1}} + \frac{{b_{0} }}{2} \le x \le \frac{{l_{{0i - 1}} + l_{{0i + 1}} }}{2}} \\ {\frac{{2a}}{{l_{{0i - 1}} - l_{{0i + 1}} - b_{0} }}x - \frac{{2a\left( {l_{{0i + 1}} + \frac{{b_{0} }}{2}} \right)}}{{l_{{0i - 1}} - l_{{0i + 1}} - b_{0} }},\frac{{l_{{0i - 1}} + l_{{0i + 1}} }}{2} < x \le l_{{0i + 1}} + \frac{{b_{0} }}{2}} \\ \end{array} } \right.$$

The calculation method of each parameter of the position function for the inner envelope is as follows:

(1) Stiffened plate spacing *li* and *l*_*i*+1_:

7 $$\begin{aligned} & li = l0i - l0i - 1 \\ & li + 1 = l0i + 1 - l0i \\ \end{aligned}$$

(2) The *x-*coordinate of the center line and distance of the center line ${l}_{ci}$:

8 $$\begin{aligned} & x0i = {(}l0i - 1 + l0i + 1{)}/2 \\ & lci = {(}li + li + 1{)}/2 \\ \end{aligned}$$

(3) Position function gradient:

9 $$\begin{aligned} & ki = \tan \alpha i = a/{(}lci - b0/2{)} \\ & \alpha i = \arctan {(}a/{(}lci - b0/2{))} \\ \end{aligned}$$

In the formula, *l*_0*i*_*,* *l*_0*i-*1_ and *l*_0*i*+1_ represent the abscissas of the three stiffening plate axis respectively.

#### Formula derivation and solution of dynamic correlation coefficient *δ*

**Figure 7:**
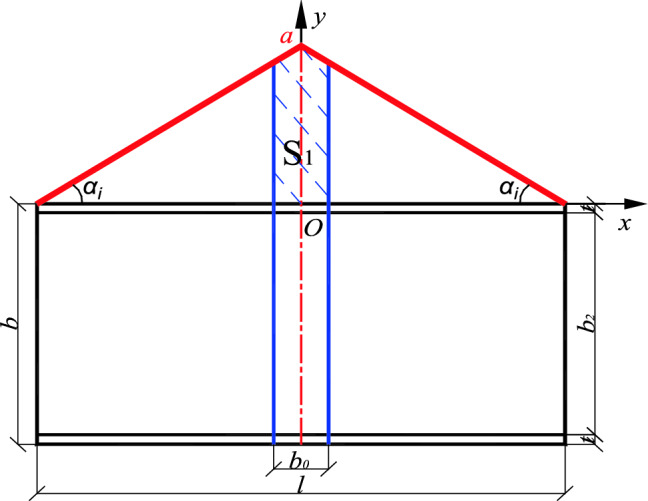
Shadow area, $${S}_{1}.$$

**Figure 8:**
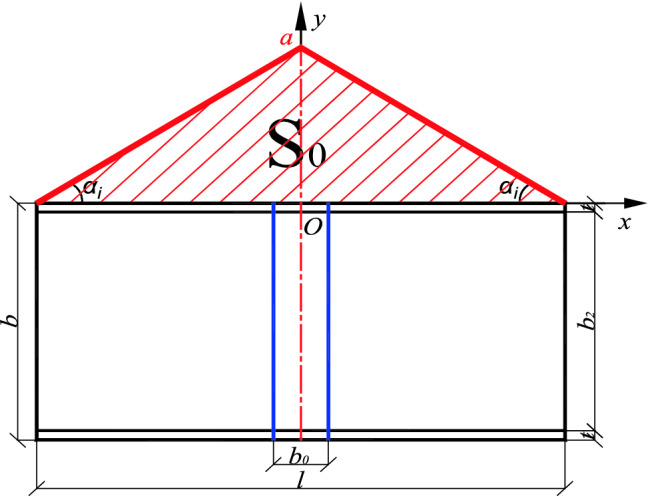
Total drawing area, ${S}_{0}.$

The dynamic correlation coefficient *δ* represents the weight of the local stiffening effect under full-length stiffening. Taking the inner envelope as an example, when a stiffened plate is positioned at the center line of the member, the dynamic correlation coefficient is equal to the ratio of the position function with the width ${b}_{0}$ of the stiffened plate to the shaded area ${S}_{1}$ surrounded by the x-axis and the general area $S_{0}$ as shown in Figs. 7 and 8. It is expressed as follows:

10 $$\delta = \frac{{S_{1} }}{{S_{0} }} = \frac{{\frac{1}{2}\left[ {\left( {\frac{l}{2} - \frac{{b_{0} }}{2}} \right) \cdot \frac{2a}{l} + a} \right] \cdot \frac{{b_{0} }}{2} \times 2}}{al/2} = \frac{{\left( {2l - b_{0} } \right) \cdot b_{0} }}{{l^{2} }}$$

(1) Dynamic correlation coefficient of the *i*th stiffened plate

**Figure 9:**
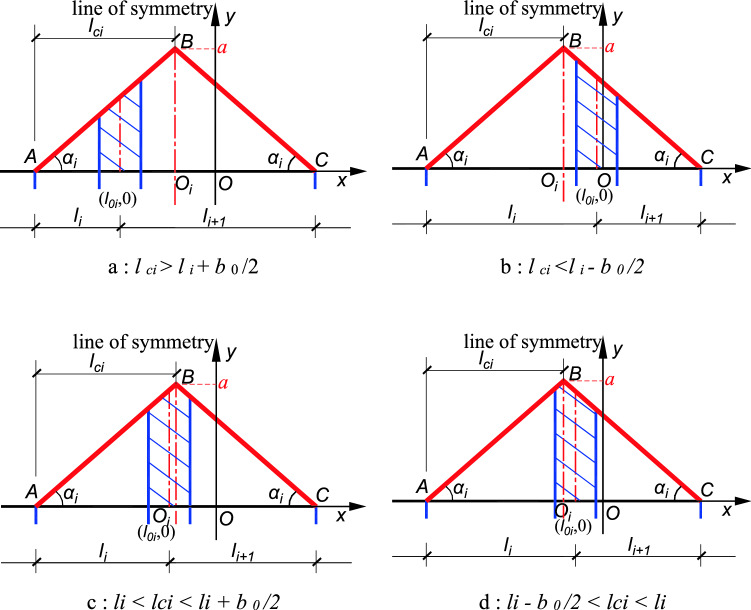
Dynamic correlation coefficient of the *i*th stiffened plate.

When a U-shaped steel sheet pile member with a length of *l* *is* fully stiffened, the whole stiffened plate can be regarded as *n* stiffened plate ($n \cdot b0 = l$) with width *b*_0._ The stiffening effect of each stiffened plate only affects the inner edges of its two adjacent stiffened plates, so the three stiffening plates are used as one unit to calculate the dynamic correlation coefficient. Taking the calculation unit of the *i*th plate as an example, the relationship between its axis and the position function line of symmetry *BO*_*i,*_ and the position of the *i*th plate in the position function interval has four situations, the specific form and the area of the shaded part are shown in Fig. 9:

The shadow area and total graphic area corresponding to the *i*th stiffened plate under different conditions are calculated as follows:

a. When/where *l*_*ci*_ > *l*_*i*_ + *b*_0_*/*2:

11 $$S_{1, i} = \left[ {\left( {l_{i} - b_{o} } \right) \cdot k_{i} + l_{i} \cdot k_{i} } \right] \cdot \frac{{b_{o} }}{2} = \left( {2l_{i} - b_{o} } \right) \cdot k_{i} \cdot \frac{{b_{o} }}{2}$$

b. When *l*_*ci*_ < *l*_*i* _*− b*_0_*/*2:

12 $$S_{1, i } = \left[ {\left( {l_{i + 1} - b_{o} } \right) \cdot k_{i} + l_{i + 1} \cdot k_{i} } \right] \cdot \frac{{b_{o} }}{2} = \left( {2l_{i + 1} - b_{0} } \right) \cdot k_{i} \cdot \frac{{b_{o} }}{2}$$

c. When *l*_*i*_ < *l*_*ci*_ < *l*_*i*_ + *b*_0_*/*2:

d. When *l*_*i*_ *− b*_0_*/*2 < *l*_*ci*_ < *l*_*i*_, the calculation formula of $S_{1, i}$ is the same.

In this case, the $S_{1, i}$ can be calculated according to the line of symmetry, divided into left and right parts, and the details are as follows:

13 $$\begin{aligned} S_{{1, i^{\prime}}} & = \frac{1}{2} \cdot \left[ {\left( {l_{i} - b_{0} } \right) \cdot k_{i} + \left( {l_{ci} - \frac{{b_{0} }}{2}} \right) \cdot k_{i} } \right] \cdot \left[ {\frac{{b_{0} }}{2} + \left( {l_{ci} - l_{i} } \right)} \right] \\ & = \frac{1}{2}\left( {l_{i} + l_{ci} - \frac{{3b_{0} }}{2}} \right) \cdot k_{i} \cdot \left( {\frac{{b_{0} }}{2} + l_{ci} - l_{i} } \right) \\ \end{aligned}$$

14 $$\begin{aligned} S_{{1, i^{\prime\prime}}} & = \frac{1}{2} \cdot \left[ {\left( {l_{i + 1} - b_{0} } \right) \cdot k_{i} + \left( {l_{ci} - \frac{{b_{0} }}{2}} \right) \cdot k_{i} } \right] \cdot \left[ {\frac{{b_{0} }}{2} + \left( {l_{ci} - l_{i} } \right)} \right] \\ & = \frac{1}{2}\left( {l_{i + 1} + l_{ci} - \frac{{3b_{0} }}{2}} \right) \cdot k_{i} \cdot \left( {\frac{{b_{0} }}{2} + l_{ci} - l_{i} } \right) \\ \end{aligned}$$

15 $$\begin{aligned} & S_{1, i} = S_{{1, i^{\prime}}} + S_{{1, i^{\prime\prime}}} \\ & \frac{1}{2}ki \cdot \left[ {\left( {l_{i} + l_{ci} - \frac{{3b_{0} }}{2}} \right) \cdot \left( {\frac{{b_{0} }}{2} + l_{ci} - l_{i} } \right) + \left( {l_{i + 1} + l_{ci} - \frac{{3b_{0} }}{2}} \right) \cdot \left( {\frac{{b_{0} }}{2} - l_{ci} + l_{i} } \right)} \right] \\ \end{aligned}$$

(2) Dynamic correlation coefficients of n (n ≥ 2) stiffening plates

When arranging *n* (*n* ≥ 2) stiffened plates, take *S*_0_ as the total area under any arbitrary stiffened member size, and the dynamic correlation coefficient is equal to the sum of area enclosed by the position function and the *x-axis* within the width of the stiffened plate b0 ($\mathop \sum \nolimits_{i = 1}^{n} S_{1, i}$, as shown in Fig. 9) The ratio to *S*_0_ is divided by the maximum value in the result, and the expression is as follows:

16 $$\delta = \frac{{\mathop \sum \nolimits_{i = 1}^{n} S_{1, i} }}{{S_{o} }} \div \frac{{max\mathop \sum \nolimits_{i = 1}^{n} S_{1, i} }}{{S_{0} }} = \frac{{\mathop \sum \nolimits_{i = 1}^{n} S_{1, i} }}{{max\mathop \sum \nolimits_{i = 1}^{n} S_{1, i} }}$$

**Figure 10:**
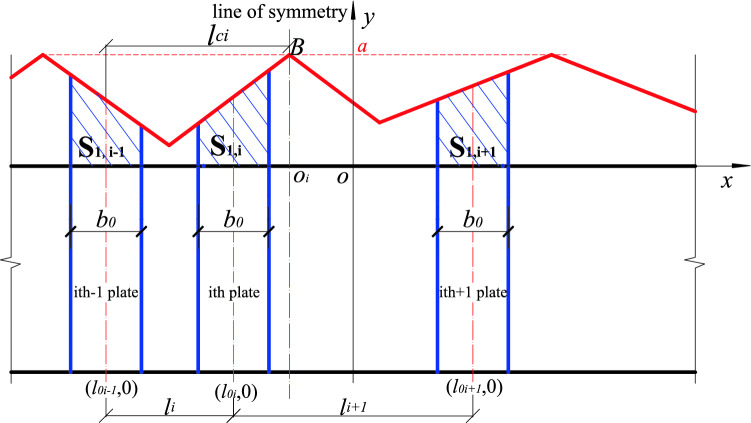
Total shaded area.

**Figure 11:**
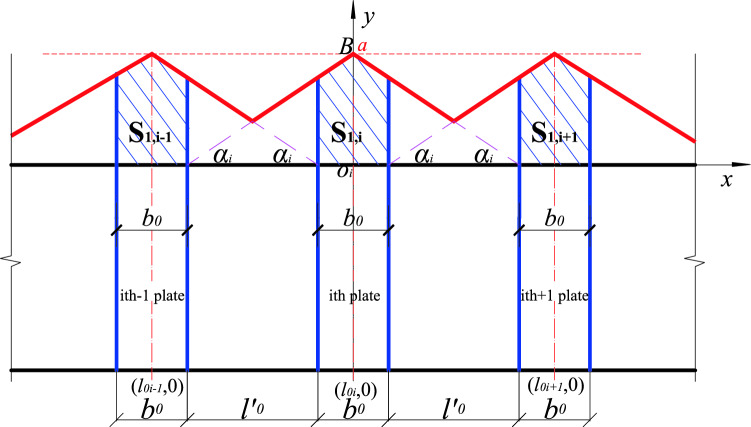
The equidistant arrangement of stiffeners.

From the general formula based on Fig. 10 shows the general working conditions of *n* pieces of stiffened plates arranged at various intervals*.* However, combined with the actual working conditions, *n* pieces of stiffened plates are arranged at equal intervals on the USSSP of length *l′*_0_ as shown in Fig. 11, the formula can be simplified as:

17 $$\begin{aligned} \delta & = \frac{{\frac{1}{2} \cdot \left[ {l^{\prime}_{0} \cdot ki + \left( {l^{\prime}_{0} + \frac{{b_{0} }}{2}} \right) \cdot ki} \right] \cdot \frac{{b_{0} }}{2} \cdot 2n}}{{S_{0} }} \div \frac{{max\mathop \sum \nolimits_{i = 0}^{n} S_{1, i} }}{{S_{0} }} \\ & = \frac{{nb_{o} \cdot \left( {2l^{{\prime}}_{0} + \frac{{b_{0} }}{2}} \right) \cdot ki}}{{2max\mathop \sum \nolimits_{i = 1}^{n} s_{1, i} }} \\ \end{aligned}$$

where is $l^{\prime}0$ the distance between the inner edges of adjacent stiffened plates, the expression is as follows:

18 $$l^{\prime}0 = \frac{l - nb0}{{n + 1}}$$

At this time, the calculation formula of the critical load value (N_max_) of the member is_:_

19 $$N_{max} = \frac{{nb_{0} \cdot \left( {2l^{{\prime}}_{0} + \frac{{b_{0} }}{2}} \right) \cdot ki}}{{2max\mathop \sum \nolimits_{i = 1}^{n} S_{1, i} }} \cdot \left( {N_{2max} - N_{1 max} } \right) + N_{1 max}$$

Combined with Fig. 3, the calculation formula of the spacing I0 of the stiffening plate is:

20 $$l0 = \frac{l + b0}{{n + 1}}$$

(3) Comparative analysis of internal and external network

The position function takes the outer envelope, the inner envelope can be used to deduce the critical load value of the stiffened member, and the calculation is as follows:

When a stiffened plate is placed at the center line of the member (*n* = 1)

21 $$N_{\max } = \frac{{\left( {2l - b0} \right)b0}}{{l^{2} }} \cdot {(}N_{2\max } - N_{1\max } {)} + N_{1\max }$$

When two stiffened plates are arranged at equal intervals (*n* = 2)

22 $$N_{\max } = \frac{{4b0 \cdot l - 2b0^{2} }}{{l^{2} + b0 \cdot l}} \cdot {(}N_{2\max } - N_{1\max } {)} + N_{1\max }$$

**Figure 12:**
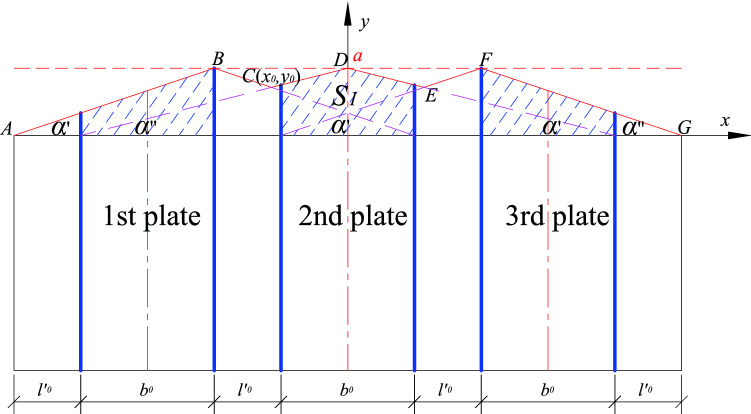
Diagram I of 3 stiffened plates arranged at equal intervals.

When *n* (*n* ≥ 3) stiffened plates are arranged at equal intervals, there are two situations, as shown in Fig. 12, when the abscissa of the intersection point C, $x0 \le 2l^{\prime}0 + b0$, the $S^{\prime}_{1}$ is calculated as follows:

23 $$\begin{aligned} S^{\prime}_{1} & = 2\left[ {\left( {b_{0} + l_{0} } \right) \cdot k^{\prime}_{i} + a} \right] \cdot \frac{{b_{0} }}{2} \cdot \frac{1}{2} \\ & = \frac{{b_{0} }}{2} \cdot \left[ {\left( {b_{0} + l_{0} } \right) \cdot k^{\prime}_{i} + a} \right] \\ \end{aligned}$$

NB: $S^{\prime}_{1}$ refers to the shaded/ shadow area corresponding to the second stiffener.

**Figure 13:**
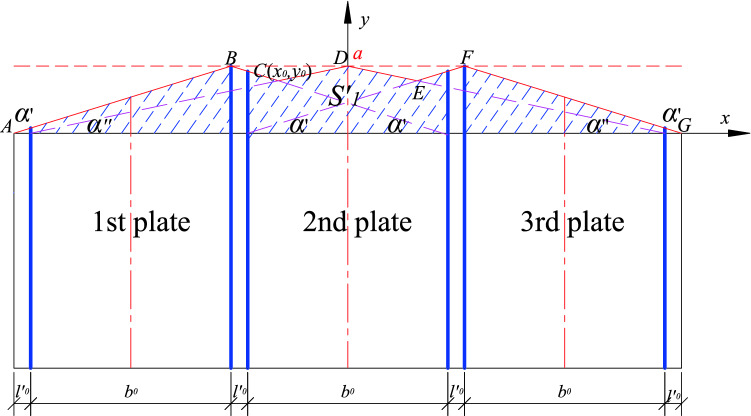
Diagram II of three stiffened plates at equal intervals.

As shown in Fig. 13, when the abscissa of the intersection point C, $x0 > 2l^{\prime}0 + b0$ the $S^{\prime}_{1}$ is calculated as follows:

24 $$\begin{aligned} S^{\prime}_{1} & = b_{0} \cdot a - 2\left[ {\frac{1}{2}\left( {\frac{{b_{0} }}{2} + l_{0} } \right) \cdot \left( {a - y_{0} } \right) - \frac{1}{2}l_{0} \cdot \left( {a - b_{0} \cdot ki} \right)} \right] \\ & = a \cdot b_{0} - \left[ {\left( {\frac{{b_{0} }}{2} + l_{0} } \right) \cdot \left( {a - y_{0} } \right) - l_{0} \cdot \left( {a - b_{0} \cdot ki} \right)} \right] \\ \end{aligned}$$

Combining the above two situations, the $\mathop \sum \limits_{i = 1}^{n} S_{1, i}$ calculation formula is as follows:

25 $$\sum_{i=1}^{n}{S}_{1, i}= \left\{\begin{array}{l}{x}_{0}\le {{2l}^{{\prime}}}_{0}+{b}_{0}\\ \sum_{i=1}^{n}{S}_{1,i}={b}_{0}\left({{l}^{{\prime}}}_{0}\cdot {k}_{i}+a\right)+\frac{\left(n-2\right){b}_{0}\left[\left({b}_{0}+{{l}^{{\prime}}}_{0}\right)\cdot {{k}^{{\prime}}}_{i}+a\right]}{2}\\ {x}_{0}>{{2l}^{{\prime}}}_{0}+{b}_{0},\\ {\sum }_{i=1}^{n}{S}_{1,i}={b}_{0}({l^{\prime}}_{0}\cdot {k}_{i}+a)+\frac{(n-3){b}_{0}\left[\left({b}_{0}+{l^{\prime}}_{0}\right)\cdot {k}_{i}+a\right]}{2}\\ +a\cdot {b}_{0}-\left[\left(\frac{{b}_{0}}{2}+{l^{\prime}}_{0}\right)\cdot \left(a-{y}_{0}\right)-{l^{\prime}}_{0}\cdot \left(a-{b}_{0}\cdot {k}_{i}\right)\right]\end{array}\right.$$

The formula for calculating the critical load value for instability is as follows:

26 $$N_{max} = \left\{ {\begin{array}{*{20}l} {x_{0} \le 2l^{{\prime}}_{0} + b_{0} ,} \hfill \\ {\frac{{\mathop \sum \nolimits_{i = 1}^{n} S_{1,i} }}{{max\mathop \sum \nolimits_{i = 1}^{n} S_{1, i} }} \cdot \left( {N_{2max} - N_{1max} } \right) + N_{1max} } \hfill \\ {x_{0} > 2l^{{\prime}}_{0} + b_{0} ,} \hfill \\ {\frac{{\mathop \sum \nolimits_{i = 1}^{n} S^{\prime}_{1,i} }}{{max\mathop \sum \nolimits_{i = 1}^{n} S^{\prime}_{1, i} }} \cdot \left( {N_{2max} - N_{1max} } \right) + N_{1max} } \hfill \\ \end{array} } \right.$$

The calculation method of each parameter in the total shadow area formula is as follows:

27 $$\begin{aligned} & l_{0}^{\prime} = \frac{l - nb_{0}}{{n + 1}} \\ & ki = \tan \alpha^{\prime} = \frac{a}{{b_{0} + l_{0}^{\prime}}} \\ & k^{\prime}i = \tan \alpha^{\prime\prime} = \frac{a}{{\frac{3}{2}b_{0} + l_{0}^{\prime}}} \\ & x_{0} = \frac{{6\left( {b_{0} + l_{0}^{\prime}} \right)^{2} }}{{5b_{0} + 4l_{0}^{\prime}}} \\ & y_{0} = \frac{{4b_{0} + 2l^{\prime}_{0}}}{{5b_{0} + 4l_{0}^{\prime}}} \\ \end{aligned}$$

Comparing Tables 3 and 4, it can be seen that the critical buckling load in the calculation results of the inner and outer envelope has a strong positive correlation with the total stiffening area. When the total stiffening area is constant, the function composed of the number of stiffened plates and critical load in the outer envelope is a monotonically decreasing function in its domain, possibly due to the linear assumption adopted in the coordination position, which needs to be further studied in combination with experiments. In most cases, there is volatility and maximum value in the function composed of the number of stiffened plates and the critical load in the inner envelope. This law conforms to the actual engineering situation and has important guiding significance for the stiffening of components.

**Table 3:** Critical load value of the inner envelope function in 10 m level component.

*l* = 10 m	n = 1	n = 2	n = 3	n = 4	n = 5	n = 6	n = 7
Stiffened area as percentage	Nmax/kN	Nmax/kN	Nmax/kN	Nmax/kN	Nmax/kN	Nmax/kN	Nmax/kN
10.0%	1332.31	1331.85	1330.93	1330.36	1329.98	1329.70	1329.50
20.0%	1436.38	1437.95	1437.06	1436.42	1435.96	1435.62	1435.36
30.0%	1528.20	1533.50	1533.42	1533.13	1532.86	1532.63	1532.45
40.0%	1607.78	1617.63	1618.87	1619.25	1619.38	1619.42	1619.42
50.0%	1675.12	1689.35	1692.12	1693.33	1693.96	1694.35	1694.60
60.0%	1730.21	1747.57	1751.64	1753.60	1754.72	1755.45	1755.96
70.0%	1773.07	1791.06	1795.63	1797.96	1799.36	1800.29	1800.95
80.0%	1803.67	1818.44	1821.98	1823.86	1825.02	1825.81	1826.39
90.0%	1822.04	1828.16	1828.16	1828.16	1828.16	1828.16	1828.16

**Table 4:** Critical load value of the outer envelope function in 10 m level component.

*l* = 10 m	n = 1	n = 2	n = 3	n = 4	n = 5	n = 6	n = 7
Stiffened area as percentage	Nmax/kN	Nmax/kN	Nmax/kN	Nmax/kN	Nmax/kN	Nmax/kN	Nmax/kN
10.0%	1332.31	1329.69	1307.52	1301.57	1298.37	1296.37	1295.00
20.0%	1436.38	1427.47	1389.62	1379.74	1374.39	1371.02	1368.72
30.0%	1528.20	1511.43	1463.39	1451.34	1444.75	1440.59	1437.72
40.0%	1607.78	1583.30	1529.71	1517.03	1510.03	1505.58	1502.50
50.0%	1675.12	1644.51	1589.33	1577.37	1570.69	1566.41	1563.43
60.0%	1730.21	1730.21	1642.90	1632.83	1627.13	1623.44	1620.85
70.0%	1773.07	1739.74	1691.14	1683.84	1679.67	1676.96	1675.03
80.0%	1803.67	1775.69	1737.46	1732.72	1730.03	1728.27	1727.04
90.0%	1822.04	1804.94	1783.10	1780.79	1779.49	1778.64	1778.04

## Analysis of Influencing Factors of Stiffening Effect on Stiffened Plates

The AU18 standard steel sheet pile with length of 10 m, 20 m, and 30 m is selected and the stiffened plate setting was carried out by using Q235 steel (*f* = 215 Mpa, *E* = 206 GPa type)*.* The influence of the stiffening effect (the number of stiffeners arranged, the width of stiffeners, the arrangement of stiffeners and the length of the components) were explored.

### The principle of the influence of single block stiffening width on the stiffening effect

**Figure 14:**
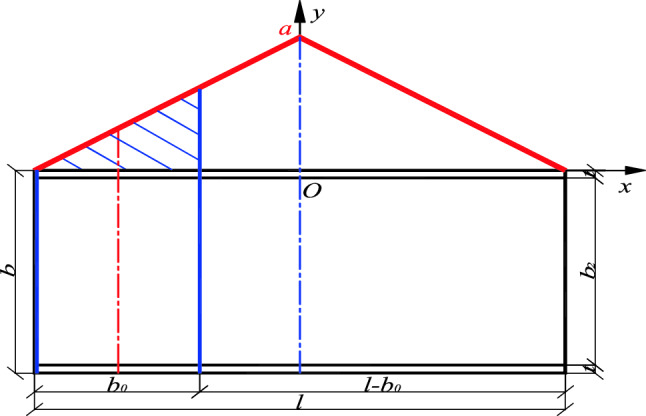
Stiffening effect of a single stiffened plate.

**Figure 15:**
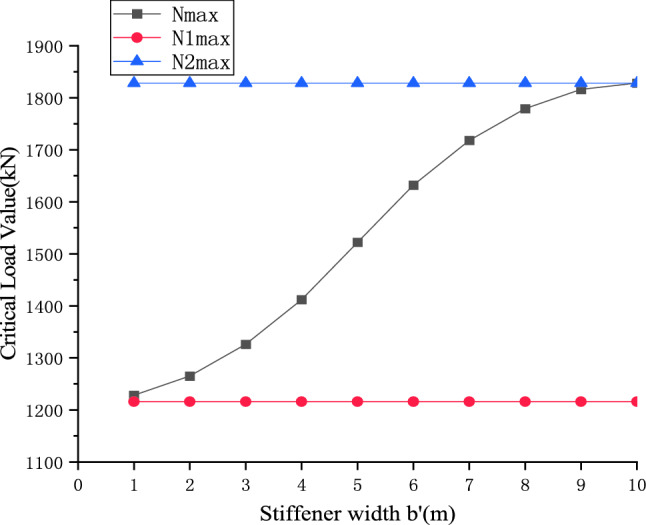
Critical load range of the stiffening member.

Taking a stiffened plate arranged with a 10 m long AU18 standard steel sheet pile as an example, the critical buckling load values of unstiffened and full length stiffened steel sheet piles calculated are 1216kN (N_1max_) and 1828.16kN (N_2max_ ) respectively (as shown in Table 2). Assuming the left end of a single stiffener is aligned with the left end of the steel sheet pile, as shown in Fig. 15, the stiffener width (*b*_0_) increases from 1 to 10 m, the critical load value (N_max_) of the member is calculated by Eq. 4, and the calculation results are shown in Figs. 14 and 15.

It can be seen from Fig. 15 that the critical load value of the member after local stiffening is between the critical load values of the unstiffened and the full-length stiffened state.

### The Influence of single block stiffener with constant width but changing position on the stiffening effect

Taking the 10 m long AU18 standard steel plate pile as an example, a single 2 m long stiffener plate is arranged, and the distance between $l^{\prime}$ the left boundary of the stiffening plate and the left boundary of the steel plate pile is defined. When the initial position is the overlap between $l^{\prime} = 0$ the left boundary of the stiffener plate and the left boundary of the steel plate pile, the position of the stiffened plate moves constantly to the right ($l^{\prime}$ continuously increasing), and the right boundary of the stiffened plate coincides with the right boundary of the steel plate pile ($l^{\prime} = 8$), as shown in Fig. 15. The change in the critical load value of the components is calculated in the process as shown in Fig. 17.

**Figure 16:**
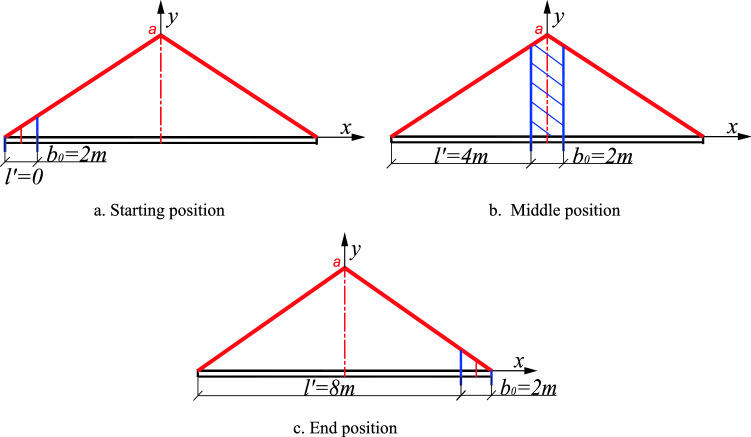
Change in stiffening position.

**Figure 17:**
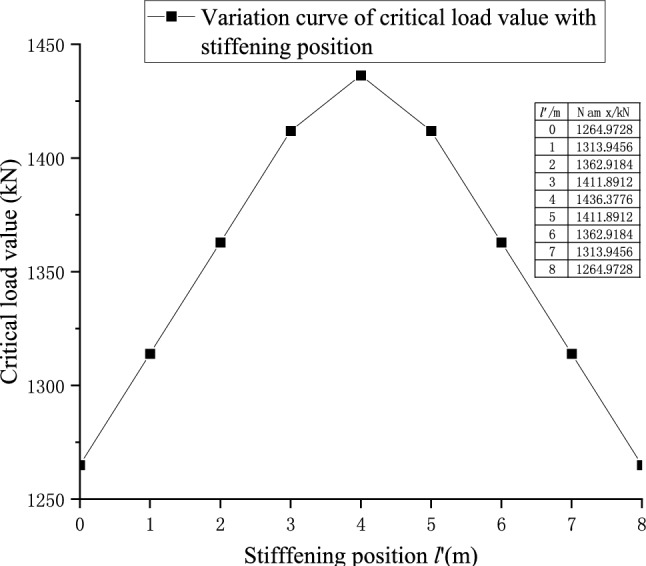
Graph of the stiffening position effect and critical load value.

From Figs. 16 and 17, when arranging the stiffened plate, the distribution of the critical load value of the member is approximately a downward opening parabola. When the stiffening position is located at the center of the motion trajectory (*l’* = 4 m), the center line of the stiffened plate coincides with the center line of the member, and the critical buckling load value of the member is the largest, and the stiffening effect is best.

### Influence of the number of stiffeners on the stiffening effects under the same stiffening area

For the 10 m, 20 m, and 30 m level members, the central arrangement of a single stiffened plate and the symmetrical arrangement of two to seven stiffened plates are used respectively to explore the relationship between the number of stiffened plates and the critical load value when the total stiffening area is equal. When the total stiffening area is constant, the arrangement scheme of the stiffened plate that maximizes the critical load value of the member, the empirical formula for the total stiffening area and the maximum critical load value in members of different lengths are derived.

28 $$y = - 14.21313x^{2} + 190.57521x + 1198.28868$$

29 $$y = - 1.00394x^{2} + 27.85063x + 386.30051$$

30 $$y = - 0.20608x^{2} + 8.58096x + 180.58223$$

The function curves of the total stiffening area *(x)* of the three members with different lengths and the critical load value *(y)* of the members are the left half of the downward opening quadratic function. With the increase of the total stiffening area, the critical buckling load values of the components increases non-linearly and tend to converge. The theoretical analysis results show that, when the total area of stiffening is unchanged, the stiffening effect gradually converges when the number of stiffeners is greater than 7. For the case of using 7 stiffeners, the relationship between the total area of different stiffeners and the critical buckling load in shown in Eqs. (28–30).

**Figure 18:**
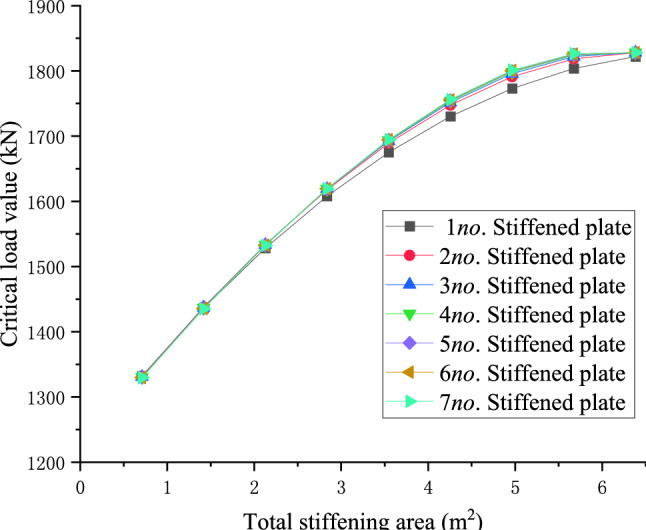
The relationship between the number of stiffened plates of 10 m level members and the critical load when the total stiffened area is the same.

**Figure 19:**
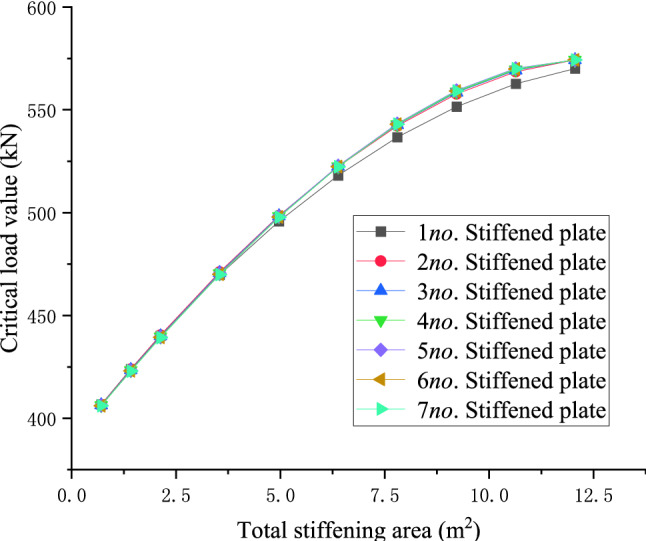
The relationship between the number of stiffened plates of 20 m level members and the critical load when the total stiffened area is the same.

**Figure 20:**
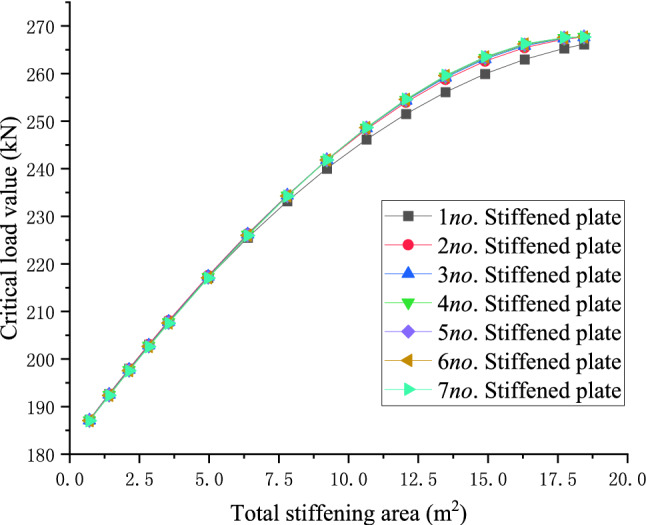
The relationship diagram between the number of stiffened plates of 30 m level members and the critical load when the total stiffened area is the same.

**Figure 21:**
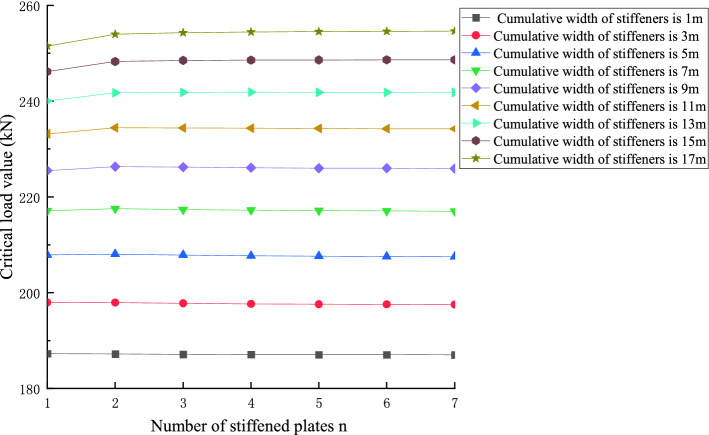
The relationship between the stiffened area and the critical load value when the number of stiffened plates of 30 m level members is the same.

**Figure 22:**
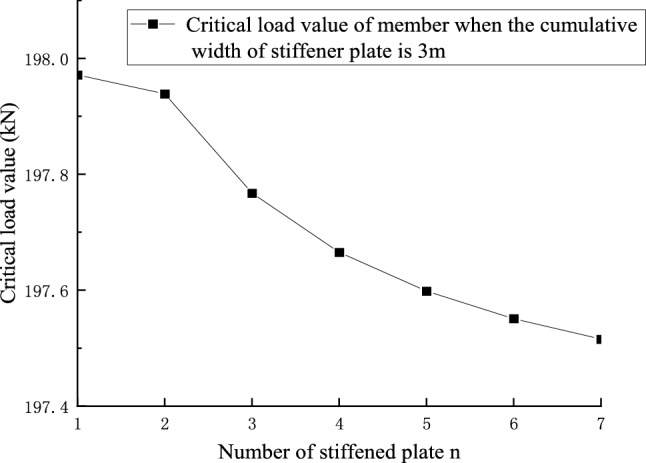
The relationship between the number of stiffened plates and the critical load value when the cumulative width of the stiffened plate of 30 m level members is 3 m.

**Figure 23:**
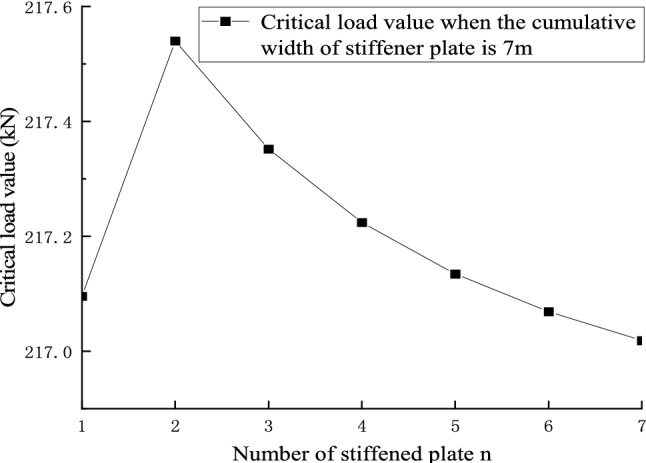
The relationship between the number of stiffened plates and the critical load value when the cumulative width of the stiffened plate of 30 m level members is 7 m.

**Figure 24:**
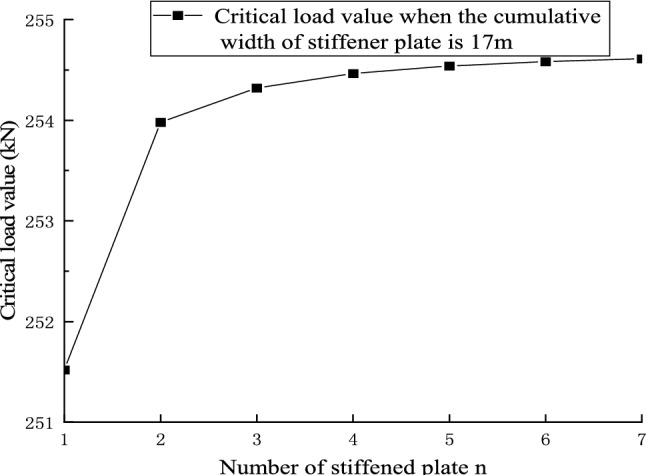
The relationship between the number of stiffened plates and the critical load value when the cumulative width of the stiffened plate of 30 m level members is 17 m.

In order to further explore the principle of the number of stiffeners, the critical load value of members, and to give the optimal layout scheme of stiffeners to improve the critical load value when the total stiffened area is known, Take a 30 m-level member as an example, the relationship between the number of stiffened plates and the critical load value of the member under different cumulative widths is shown below:

Figures 22 and 23 shows the effect of non-positive correlation between the critical load value of member instability and the number of stiffened plates, because the width of the stiffened plates is too small, resulting in local instability. Through the comparative analysis of Figs. 18, 19, 20, 21, 22, 23 and 24 it can be seen that the critical load value of the member is positively correlated with the total stiffening area, and the stiffened plate arrangement scheme that maximizes the critical load value of the member is obtained when the total stiffening area is constant. The results are summarized in Table 5.

**Table 5:** Optimal number of stiffened panels under different total stiffened area.

*l* = 10 m	n = 1	n = 2	n = 3	n = 4	n = 5	n = 6	n = 7
Percentage of stiffened area	10–13%	14–30%	31–34%	35–37%	38%	39–40%	40–80%
Cumulative width of stiffeners/m	1–1.3	1.4–3	3.1–3.4	3.5–3.7	3.8	3.9–4	4–8
*l* = 20 m	n = 1	n = 2	n = 3	n = 4	n = 5	n = 6	n = 7
Percentage of stiffened area	5–8%	10–35%	none	none	35.5–75%	none	none
Cumulative width of stiffeners/m	1–1.9	2–7	none	none	7.1–15	none	none
*l* = 30 m	n = 1	n = 2	n = 3	n = 4	n = 5	n = 6	n = 7
Percentage of stiffened area	3.3–11.7%	12–38%	38.3–42.3%	42.7–45%	45.3–46.7%	46.7–48%	48–83.3%
Cumulative width of stiffeners/m	1–3.5	3.6–11.4	11.5–12.7	12.8–13.5	13.6–14	14–14.4	14.4–25

For the 10 m USSSP stiffened plate with a cumulative width of 1–1.3 m, and the total stiffening effect of the stiffened plate is 1–3 m, the best effect is achieved when a single stiffener is arranged at the center. Meanwhile when the cumulative width of the stiffening plate is 1.4–3 m, the equal interval layout of two (2) stiffened plates is the best when the width is 3.1–4 m, 3–6 stiffeners can be arranged at equal intervals according to the actual situation. When the cumulative width of the stiffeners is 4–8 m, the best effect is to arrange 7 stiffeners at equal intervals. When it reaches 8 m, the critical load of the overall instability of the component is the largest.

For the 20 m grade USSSP stiffened plate cumulative width of 1–1.9 m, the single stiffener plate effect is the best. When the cumulative width of the stiffener is 2–7 m, it is best to arrange two (2) stiffeners at equal intervals. With the cumulative width of stiffened plate 7.1–15 m, equal spacing arrangement of 5 stiffened plate effect is the best. When the cumulative width of 5 pieces of stiffened plate of the size reaches 15 m, the overall instability critical load of the member is the largest.

For the 30 m USSSP stiffened plate cumulative width of 1–3.5 m, the center layout of a single stiffened plate effect is the best. When the cumulative width of the stiffened plate is 3.6–11.4 m, equal spacing arrangement of two stiffened plate effect is the best. When the cumulative width of the stiffened plate is 11.5–14.4 m, you can choose the same size of the equal spacing layout of 3–6 stiffened plate according to the actual situation. When the cumulative width of the stiffeners is 14.4–25 m, the best effect is to arrange seven stiffeners at equal intervals. When the cumulative width of 7 stiffened plates of the same size reaches 25 m, the overall instability critical load of the component is the greatest.

## Conclusion

USSSP have bending and buckling problems in the engineering projects. In order to resolve this problem, stiffened plates are set along the longitudinal direction of USSSP and the dynamic correlation coefficient is proposed according to the stress characteristics of the USSSP during the dynamic piling process. The method of calculating the critical buckling load of USSSP after local stiffening is established. The bending stiffness of steel sheet piles can be improved by changing the basic properties and conditions of the locking systems. The critical buckling load value of USSSP after stiffening can be obtained more effectively and quickly using this method, which can be used to optimize the longitudinal stiffening arrangement of USSSP and verify the range of critical load values after stiffening.

Taking 10 m, 20 m, and 30 m grade AU18 standard USSSP as examples, according to the arrangement of stiffened plates, the total stiffening area and the quantitative number of factors. The regular analysis of the critical load of the overall instability of the components is carried out, and the results are given when the total stiffening area is constant. The layout plan of the number of stiffening plates that maximizes the critical load value of the components of the total stiffening is derived. This verifies the rationality of the calculation method of the critical buckling load of the locally stiffened USSSP based on the idea of the dynamic correlation coefficient method, and solves the instability problem of the application of the axial compression members in engineering application. The instability problem in the model provides a theoretical basis for the local stiffening of such components, but the linear assumption is used when allocating weights, which is somewhat complicated and needs to be further researched.

## Data Availability

On reasonable request, the corresponding author will make some of the models and data that support the study available.
